# Vocabulary and Functional Communication in Preschool‐Aged Children With Cochlear Implants

**DOI:** 10.1002/brb3.71664

**Published:** 2026-08-17

**Authors:** Aysun Parlak Kocabay, Merve İkiz Bozsoy, Esra Yücel

**Affiliations:** ^1^ Department of Audiology Hacettepe University Ankara Turkey; ^2^ Department of Speech and Language Therapy Anadolu University Eskisehir Turkey

**Keywords:** age at implantation, cochlear implantation, functional communication, vocabulary development

## Abstract

**Objective:**

Early cochlear implantation is a critical factor influencing spoken language development in children with congenital hearing loss. Vocabulary acquisition, as a core component of language, is dependent on early auditory access. This study aimed to examine the relationship between functional auditory communication and receptive–expressive vocabulary development in children aged 2–6 years with cochlear implants, and to compare outcomes according to age at implantation.

**Methods:**

This retrospective study included 29 children with cochlear implants aged between 2 and 6 years. Participants were divided into two groups based on age at first implantation: 12–18 months (*n* = 16) and 19–24 months (*n* = 13). Functional communication was assessed using the Functioning after Pediatric Cochlear Implantation (FAPCI). Vocabulary development was evaluated with the Turkish Expressive and Receptive Vocabulary Test (TIFALDI). Group comparisons were performed and correlations between functional communication and vocabulary measures were examined.

**Results:**

Children implanted between 12 and 18 months demonstrated significantly higher functional communication outcomes, including FAPCI total (*p* = 0.04), speech perception (*p* = 0.04), and speech production scores (*p* = 0.032) compared to those implanted between 19 and 24 months. Similarly, significantly higher receptive (*p* = 0.001) and expressive (*p* = 0.003) vocabulary scores were observed in the earlier‐implanted group. Strong positive correlations were found between receptive vocabulary and FAPCI total (*r* = 0.734) and speech perception scores (*r* = 0.689), while expressive vocabulary showed strong to very strong correlations with FAPCI total (*r* = 0.701) and speech production scores (*r* = 0.765) (*p* < 0.001).

**Conclusion:**

Earlier cochlear implantation within the first 18 months of life is associated with superior functional communication and vocabulary outcomes in early childhood. The strong associations between functional auditory communication and receptive–expressive vocabulary highlight the central role of lexical development in spoken language outcomes following cochlear implantation.

## Graphical abstract

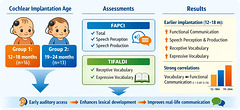

## 1 Introduction

Human hearing begins developing in utero: by approximately the 23rd week of gestation the cochlea is functional (Arat et al. 2024). This early auditory access enables infants to acquire spoken language and cognitive skills through listening, laying the foundation for later literacy and academic success. In children with typical hearing, the expressive lexicon grows rapidly during the second year of life; parental checklist data indicate that toddlers produce around 50 words by 18 months and over 300 words by 24 months (Eshghi et al. 2019). This “vocabulary spurt” often coincides with the onset of two‐word combinations and the emergence of basic grammar (Eshghi et al. 2019). Cross‐linguistic research reveals that early vocabularies are dominated by open‐class words such as nouns and verbs; as children's lexical inventories expand beyond 50–100 words, adjectives and function words begin to appear (Marjanovič‐Umek et al. 2013).

Vocabulary development does not occur in isolation: it is closely tied to phonological processing, morphosyntactic growth and later reading ability. Children with larger early vocabularies tend to show more advanced phonological awareness and grammatical skills and achieve better literacy outcomes in school (Lund 2016). Conversely, diminished lexical knowledge is associated with delays in these domains. Because vocabulary is such a sensitive indicator of broader language development, a detailed understanding of early lexical growth can provide speech‐language therapists and educators with valuable cues regarding a child's need for intervention.

For children born with severe or profound hearing loss, the trajectory of vocabulary development is fundamentally altered. Cochlear implants (CIs) now offer a widely accepted means of providing auditory access, and neonatal hearing screening ensures that many infants with congenital hearing loss are identified early enough to receive timely intervention (Arat et al. 2024). Nevertheless, children with CIs do not typically receive auditory input until at least 12 months of age because regulatory guidelines usually restrict surgery before that time (Lund 2016). As a result, they begin acquiring spoken language later than their hearing peers, and outcomes regarding whether they ultimately achieve age‐appropriate vocabularies remain mixed. Longitudinal studies have found that children implanted before 30 months can achieve receptive vocabulary scores within the normal range by age six, whereas other findings suggest that many children with CIs remain behind their hearing peers throughout childhood, despite early implantation. Such discrepancies are likely due to a complex interplay of factors, including age at surgery, duration of device use, quality of auditory rehabilitation, communicative mode in the home and school environments, and general cognitive abilities. Even under favorable conditions, vocabulary growth must accelerate substantially for CI users to “catch up,” as they need to learn words at a faster rate than their hearing peers in order to close the lexical gap (Busch et al. 2022; Lund 2016; Wie et al. 2020).

Much of the existing research relies on global vocabulary measures that provide limited insight into how well children apply their auditory skills in daily contexts. While receptive and expressive vocabulary scores offer broad estimates of lexical development, they do not fully capture children's ability to recognize and use words in real‐life settings. Measures of functional hearing, which assess listening behaviors in everyday situations, therefore provide complementary information and may shed light on the relationship between auditory access and vocabulary knowledge. Recent findings suggest that children with higher functional hearing scores exhibit stronger overall language skills, but most studies have not examined whether this association extends specifically to receptive and expressive vocabulary development (Arat et al. 2024). Considering this, the present study investigates the relationship between functional hearing and both receptive and expressive vocabulary in children aged 2–6 years who use cochlear implants. By combining a vocabulary‐focused assessment with a parent‐reported measure of everyday listening performance, we aim to determine whether children with greater auditory functionality possess richer lexical knowledge. Clarifying the relationship between functional communication and vocabulary development during the preschool years may inform clinical monitoring strategies and early intervention planning in children with cochlear implants.

## 2 Materials and Methods

All procedures were conducted in accordance with the ethical principles outlined in the Declaration of Helsinki (JAMA, 2000; 284:3043–3049), and ethical approval was obtained from the Anadolu University Health Science Ethics Board (approval code: 926614). Written informed consent was obtained from the parents or legal guardians of children. This was a retrospective cohort study based on clinical data.

### 2.1 Participants

All participants received their CI at Hacettepe University with a surgical technique by a small incision described in detail previously. Cochlear implant devices from Cochlear and MED‐EL were used according to clinical indication and availability. Full insertion of the electrode array was confirmed in all patients postoperatively by transorbital X‐ray. No postoperative surgical complications were observed in the study cohort. Following implantation, all children attended regular mapping sessions and routine clinical follow‐up at our center.

A total of 29 children with cochlear implants (CIs), aged between 2 and 6 years, were included in the study. The inclusion criteria were as follows: (1) having received their cochlear implants at Hacettepe University Hospital, (2) bilateral congenital severe‐to‐profound sensorineural hearing loss, (3) consistent follow‐up for implant mapping at our center, (4) regular participation in auditory rehabilitation for a minimum of 2 h per week, and (5) aided sound‐field thresholds with CIs falling within the speech frequency–intensity range. Children with inner ear malformations, auditory neuropathy spectrum disorder, neurological or developmental conditions, learning disabilities, or other comorbidities were excluded. All participants were native Turkish‐speaking children and were raised in Turkish‐speaking home environments.

Based on the age at first implantation, participants were categorized into two groups: those implanted between 12 and 18 months (*n* = 16; 9 females, 7 males) and those implanted between 19 and 24 months (*n* = 13; 7 females, 6 males). Of the 29 participants, 18 were bilateral cochlear implant users and 11 were unilateral cochlear implant users. No significant differences were observed between the groups in terms of parental education level or the distribution of unilateral versus bilateral CI users. Participant demographics are shown in Table 1.

**TABLE 1:** Demographics of the subjects.

	Age (m)	Implantation age (m)	Hearing aided age (m)	Gender (*n*, %)		Parental education level (*n*, %)		
				F	M	Primary school	High school	Bachelor degree
**12–18 CI (*n* = 16)**	51.3 ± 13.1	14.7 ± 1.8	6.8 ± 2.1	9 (56)	7 (44)	5 (31)	7 (44)	4 (25)
**19–24 CI (*n* = 13)**	54.4 ± 11.7	21.4 ± 1.6	8.2 ± 2.5	7 (54)	6 (46)	6 (46)	5 (39)	2 (15)

### 2.2 Functioning After Pediatric Cochlear Implantation (FAPCI)

The Functioning after Pediatric Cochlear Implantation (FAPCI) is a family‐centered scale designed to assess communicative performance, grounded in the World Health Organization's conceptual framework of functioning. Unlike measures focusing solely on language skills, the FAPCI evaluates verbal communication within educational and behavioral contexts. Developed in 2007 by Lin et al., this tool was originally created to provide an objective evaluation of auditory performance in children with cochlear implants. The scale consists of 23 items covering auditory environment, receptive communication, expressive communication, and socialization, with total scores ranging from 23 to 115 Kutlu 2021. The FAPCI provides information not only on overall functional communication but also on speech perception and speech production abilities. Specifically, items 4, 5, 7, 8, 9, 14, and 16 assess speech production, whereas items 1, 2, 13, 18, 19, and 20 evaluate speech perception. The Turkish adaptation and validity and reliability study of the scale, was conducted by Özkan et al. (2020).

### 2.3 Turkish Expressive and Receptive Vocabulary Test (TIFALDI)

The Turkish Expressive and Receptive Vocabulary Test (TIFALDI) is a vocabulary assessment tool developed specifically for Turkish‐speaking children and standardized by Güven and Berument. TIFALDI was created based on the Turkish language structure and standardized on a large, nationally representative sample, ensuring its validity and reliability. The receptive vocabulary subtest comprises 104 target words, while the expressive subtest includes 80 target words (Berument and Güven 2013). The receptive vocabulary subtest requires children to identify target words from picture arrays, whereas the expressive vocabulary subtest requires them to verbally name the presented items.

Statistical analyses were conducted using the standard scores obtained from the receptive and expressive subtests. Standard scores, calculated by transforming raw scores relative to the norm group, allow for objective comparisons across individuals and groups.

### 2.4 Data Analysis

Statistical analysis was performed using SPSS 26 (IBM Corp). Descriptive statistics were presented as mean, standard deviation, and minimum–maximum values. The normality of the data distribution was assessed with the histograms, probability plots, Kolmogorov‐Smirnov and Shapiro–Wilk tests. To compare the scores of the two implantation age groups (12–18 CI vs. 19–24 CI), the Independent Samples *t*‐test was used for normally distributed variables, whereas the Mann–Whitney *U* test was applied when normality was not satisfied. Relationships between variables were examined using the Pearson correlation coefficient for normally distributed data, or the Spearman's correlation coefficient when the normality assumption was not met. A significance level of *p* < 0.05 was considered for all analyses. The strength of correlation was interpreted as follows: *r* < 0.20 = very weak or no correlation; 0.20–0.40 = weak; 0.40–0.60 = moderate; 0.60–0.80 = strong; and *r* > 0.80 = very strong correlation.

## 3 Results

**FIGURE 1:**
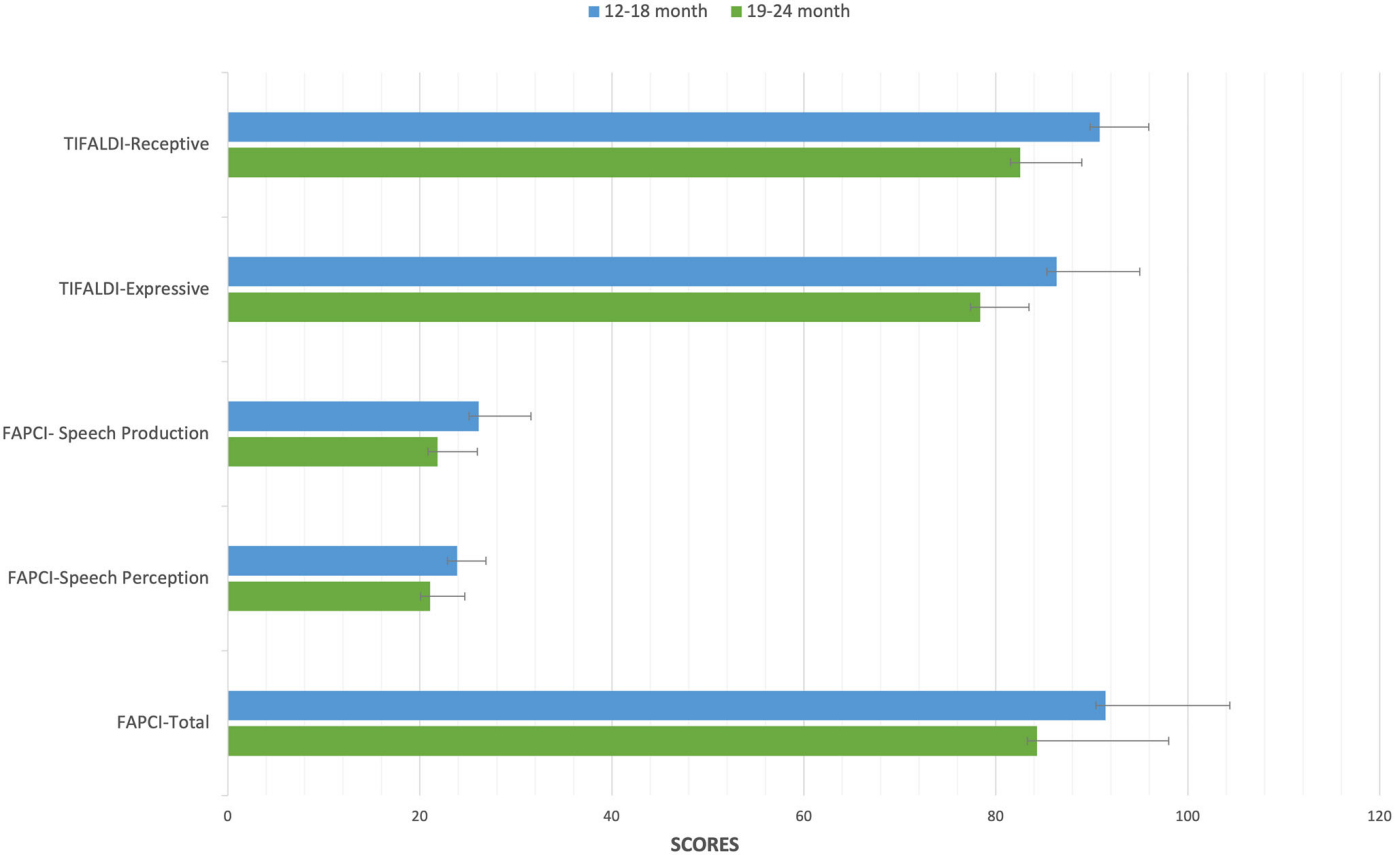
Comparison of mean FAPCI total, FAPCI speech perception, FAPCI speech production, and TIFALDI receptive and expressive vocabulary scores between children implanted at 12–18 months and 19–24 months. Error bars indicate ±1 standard deviation.

Children implanted between 12 and 18 months demonstrated significantly higher FAPCI total scores (*p* = 0.04), speech perception scores (*p* = 0.04), and speech production scores (*p* = 0.032) compared to those implanted between 19 and 24 months. Group comparisons of mean scores (± SD) for FAPCI and TIFALDI outcomes are illustrated in Figure 1.

Similarly, significant group differences were observed in vocabulary outcomes measured by TIFALDI. Children implanted between 12 and 18 months showed significantly higher scores in both the receptive vocabulary subtest (*p* = 0.001) and the expressive vocabulary subtest (*p* = 0.003) than those implanted between 19 and 24 months (Figure 1).

**FIGURE 2:**
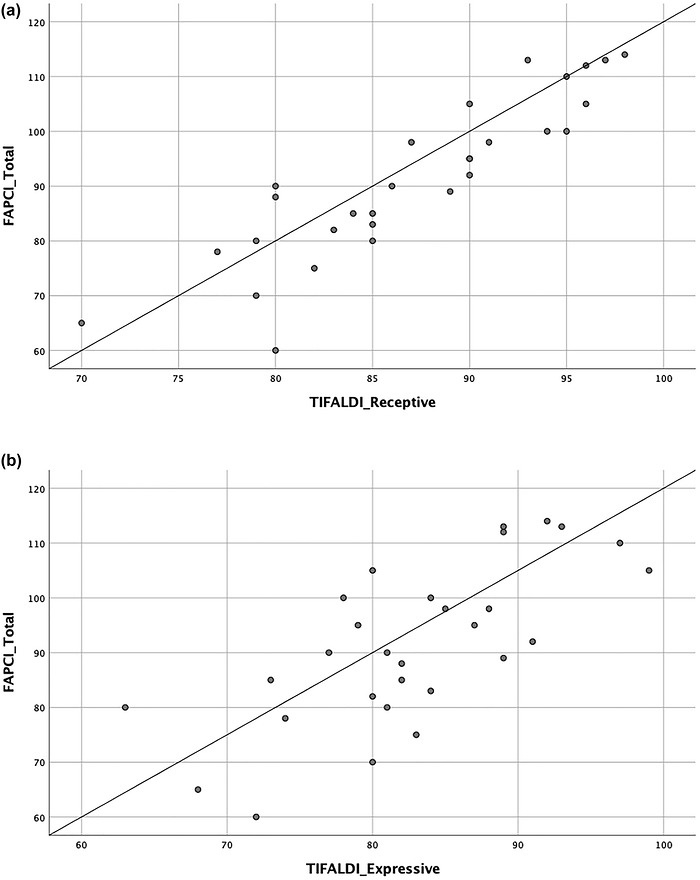
Relationship between FAPCI total scores and TIFALDI receptive (a) and expressive (b) vocabulary scores.

Correlation analyses revealed strong positive associations between vocabulary and functional communication measures. As shown in Figure 2, TIFALDI receptive vocabulary scores showed a strong correlation with FAPCI total scores (*r* = 0.734, *p* < 0.001) and with FAPCI speech perception scores (*r* = 0.689, *p* < 0.001).

**FIGURE 3:**
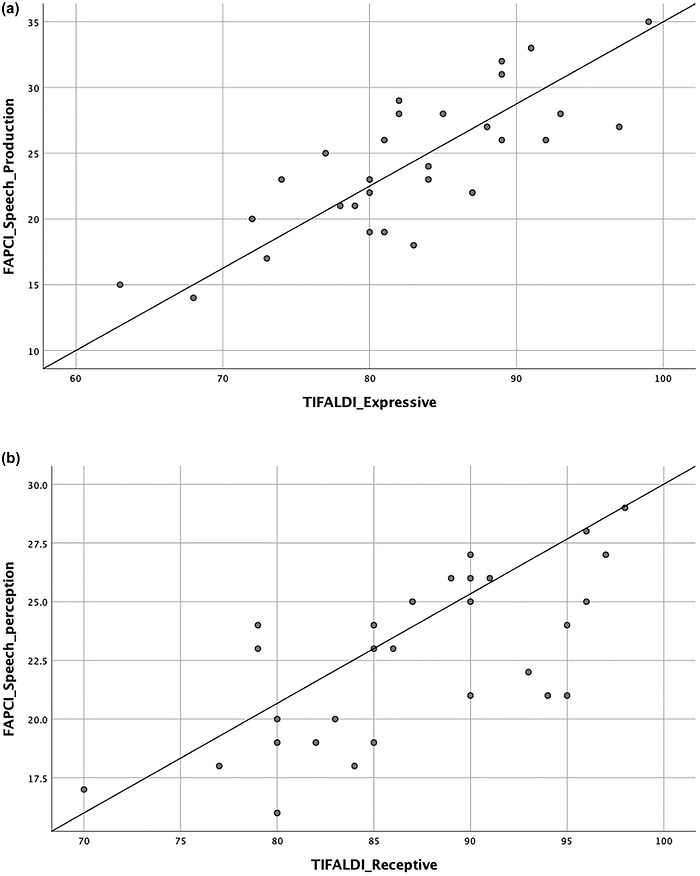
Relationship between FAPCI speech perception and TIFALDI receptive vocabulary scores (a) and relation between FAPCI speech production and TIFALDI expressive vocabulary scores (b).

As shown in Figure 3, TIFALDI expressive vocabulary scores were strongly correlated with FAPCI total scores (*r* = 0.701, *p* < 0.001) and FAPCI speech production scores (*r* = 0.765, *p* < 0.001).

## 4 Discussion

The present study investigated the relationship between functional auditory communication and vocabulary development in children with cochlear implants aged 2–6 years, with a particular focus on age at implantation. By integrating a parent‐reported functional communication measure with a standardized receptive and expressive vocabulary assessment, this study provides a contemporary and clinically relevant perspective on early communicative outcomes following cochlear implantation.

Consistent with recent evidence, children who received cochlear implants between 12 and 18 months demonstrated significantly better outcomes than those implanted between 19 and 24 months across both functional communication and vocabulary measures. Recent large‐scale and longitudinal studies have emphasized that implantation within the first 2 years of life, and particularly before 18 months, is associated with more favorable language trajectories (Ching et al. 2025; Chu et al. 2020; Duchesne and Marschark 2019). The present findings support and extend this evidence by demonstrating that these advantages are evident not only in vocabulary measures but also in everyday communicative functioning.

Significant group differences were observed in both receptive and expressive vocabulary outcomes as measured by TIFALDI. Given that TIFALDI primarily assesses vocabulary rather than broader syntactic or morphosyntactic skills, these results highlight the sensitivity of lexical development to early auditory access. Recent research has shown that early cochlear implantation supports more efficient word learning by enhancing access to phonological detail during a critical period for lexical mapping (Jung et al. 2020). Children implanted earlier may therefore experience richer and more consistent opportunities for vocabulary acquisition during daily interactions.

In parallel, children implanted between 12 and 18 months demonstrated higher FAPCI total, speech perception, and speech production scores. FAPCI captures functional communication in real‐life listening environments and has been increasingly used to complement language testing in pediatric CI research (Lin et al. 2007; Ostojić et al. 2015). The present findings suggest that earlier auditory access supports not only linguistic knowledge but also the effective use of hearing for communication in everyday contexts, reinforcing the validity of functional outcome measures.

The strong correlations observed between TIFALDI and FAPCI scores further underscore the close relationship between vocabulary development and functional auditory communication. Receptive vocabulary was strongly associated with functional communication and speech perception, whereas expressive vocabulary showed the strongest association with speech production. Recent models of spoken language development in children with hearing loss emphasize that vocabulary growth serves as a central link between auditory perception and spoken language use (Duchesne and Marschark 2019; Majorano et al. 2021). The present findings align with these models, suggesting that functional hearing abilities and lexical development evolve in an interdependent manner. Taken together, these findings suggest a developmental pathway whereby earlier cochlear implantation provides earlier access to auditory input, facilitating vocabulary acquisition during a critical period of language development. As vocabulary knowledge expands, it enables children to recognize, comprehend, and produce spoken language more efficiently during everyday interactions.

From a clinical perspective, these results highlight the importance of incorporating both functional communication measures and vocabulary‐based assessments when evaluating outcomes in young children with cochlear implants. While functional tools such as the FAPCI provide valuable insight into real‐world communication, vocabulary assessments capture foundational linguistic knowledge that predicts later grammatical development, academic achievement and literacy (Davidson et al. 2019).

The present study has several limitations. First, the retrospective design and relatively small sample size may limit the generalizability of the findings. Second, the study relied on existing clinical records; therefore, potentially influential factors such as the quality and quantity of language input in the home environment, parental interaction styles and socioeconomic standards, objective measures of daily cochlear implant use (e.g., datalogging), and cognitive abilities could not be systematically controlled. Third, TIFALDI primarily assesses receptive and expressive vocabulary and does not capture the separate type of vocabulary and broader linguistic domains. Fourth, the absence of an age‐matched normal‐hearing control group limits the ability to contextualize the extent of vocabulary delay or catch‐up following cochlear implantation. Finally, functional communication outcomes were assessed using a parent‐reported measure, which may be influenced by parental perceptions. Future prospective longitudinal studies with larger samples and more comprehensive language measures are needed to further elucidate the developmental pathways linking functional communication and vocabulary growth in children with cochlear implants.

## 5 Conclusion

The present study demonstrates that cochlear implantation between 12 and 18 months of age is associated with significantly better functional communication and vocabulary outcomes in children aged 2–6 years compared to implantation between 19 and 24 months. The strong associations observed between FAPCI scores and receptive and expressive vocabulary outcomes highlight the close interdependence of functional auditory abilities and lexical development during early childhood. These findings underscore the importance of early auditory access and support the use of combined functional and vocabulary‐based assessments to obtain a comprehensive understanding of communicative outcomes in young children with cochlear implants. These results reinforce the importance of early implantation and comprehensive early intervention supporting functional communication and language development.

## Author Contributions

**Merve İkiz Bozsoy**: investigation, methodology, data curation. **Aysun Parlak Kocabay**: writing – original draft, writing – review and editing, conceptualization, formal analysis. **Esra Yücel**: supervision, methodology.

## Funding

This study is supported by TUBITAK (Türkiye Bilimsel ve Teknolojik Araştırma Kurumu).

## Conflicts of Interest

There are no conflicts of interest, financial or otherwise.

## Data Availability

Data available on request due to ethical restrictions.
